# Subtidal kelp habitat classification at the Isles of Shoals: An integrative Random Forest approach using bathymetry and Landsat imagery

**DOI:** 10.1371/journal.pone.0338218

**Published:** 2025-12-18

**Authors:** Constance N. Tyler, Kim Lowell, Christopher E. Parrish, Jennifer A. Dijkstra

**Affiliations:** 1 Department of Biological Sciences, University of New Hampshire, Durham, New Hampshire, United States of America; 2 Center for Coastal and Ocean Mapping/Joint Hydrographic Center, Durham, New Hampshire, United States of America; 3 College of Engineering, Oregon State University, Corvallis, Oregon, United States of America; Swedish University of Agricultural Sciences and Swedish Institute for the Marine Environment, University of Gothenburg, SWEDEN

## Abstract

Kelps form ecologically important habitats around the globe but are threatened by anthropogenic stressors in much of their range. Within the Gulf of Maine, these stressors include rising ocean temperatures and species invasions. Monitoring these habitats is important, but our ability to do so varies regionally based on kelp species. Modelling techniques based on optical satellite imagery are useful for floating kelps but can only identify the subsurface kelps found in the Gulf of Maine within a small upper portion of their depth range. We developed an integrative approach to kelp habitat classification using two existing data sources: sea surface temperature data from Landsat 8 and high-resolution acoustic bathymetry data in a 10-by-13 km area around the Isles of Shoals. Ground truth data were collected by lowering and raising cameras from the seabed; observations were divided into bare substrate, kelp habitat, red turf macroalgae habitat, and intermediate “mixed” macroalgae habitat classes, and used to train a Random Forest model. The model classified benthic habitats with 71% accuracy. Depth, median summer sea surface temperature, vector ruggedness measure, and slope were among the most important variables in classifying kelp habitat. This approach improves upon previous modelling and monitoring methods by expanding the depth range and total amount of area that can be assessed, while also addressing the importance of temperature in mediating substrate competition between kelps and other macroalgae. It may be generalizable to the Gulf of Maine and to other regions where kelp habitats face similar stressors and may aid in identifying healthy habitats for conservation.

## Introduction

Kelps provide important biogenic habitat worldwide. They act as refuge for a wide variety of marine organisms, including marine mammals [1,2], sharks [3], bony fish [4], and crustaceans [5,6]. There is ongoing research into the role of kelp detritus in carbon cycling, and how these carbon sequestration services may be enhanced [7–9]. Possibilities for the expansion of kelp mariculture efforts are also being investigated across many regions [10–12].

In the Gulf of Maine, kelp habitats (i.e., locations where kelps are the dominant form of macroalgal cover) are affected by several anthropogenic stressors. Multiple marine species introductions have contributed to changes in benthic community composition and distribution in recent decades [13,14]. The loss of kelp beds coincides with the expansion of “red turf” habitats. These turfs are formed by assemblages of filamentous red macroalgae, especially the introduced species *Dasysiphonia japonica* [15,16], and their dominance reshapes the three-dimensional structure of benthic habitats [4,17,18]. The Gulf of Maine is warming more quickly than 99% of global seas [19], and increasing intensity and frequency of marine heatwaves are expected to have long-term impacts on kelp performance [20].

Monitoring large benthic areas is an important task in the conservation, management, and restoration of kelp habitats facing these (and other) threats, but monitoring is faced with logistical challenges. Observation of subsurface kelp communities has traditionally relied on surveys by SCUBA divers, but these surveys require intense effort and can only cover small fractions of total benthic area. Within the areas studied, surveys often make use of small quadrats (<1 m^2^) or transect methods that do not guarantee comprehensive coverage. These surveys are also frequently restricted to the upper portions of kelp depth ranges – recent SCUBA surveys in the Gulf of Maine do not exceed 15 m depth [15,17,21], while kelp habitats can occur to depths greater than 25 m [22,23]. While floating, forest-forming kelps such as those found in the Northeast Pacific, South America, and New Zealand can be quantified using optical imagery from aerial and satellite surveys [24–29], such techniques are thus far only effective at modelling the subsurface kelp beds present in the North Atlantic to depths of 7 m [30]. Studies using acoustics to map kelp habitats are less constrained by depth (or optical depth) than optical models, and can cover larger areas than SCUBA divers [31–33], but may not be appropriate in all regions. The pattern of kelp decline in the Gulf of Maine is one where kelps are lost and then competitively excluded from substrate matching their bathymetric preferences; models must account for additional factors such as water temperature that are likely to mediate competition between these community types. Researchers in Brittany, France used a modelling approach integrating bathymetry and sea surface temperature (SST) data to identify kelp habitats [34]. This SST data was produced by the Advanced Very-High-Resolution Radiometer at a resolution of 1.1 km – the coarse resolution greatly limited the ability of the researchers to make local-scale predictions of kelp habitat. Following the launch of Landsat 8 in February 2013, the Landsat Program began to offer SST data at 30 m spatial resolution [35].

Here, we developed a Random Forest (RF) approach to kelp habitat modelling that integrates SST data from Landsat 8, high-resolution multibeam echosounder bathymetry data, and benthic surface characteristics derived from this bathymetry data. The use of Landsat-derived SST data in marine habitat modelling is minimal thus far, though the data have been used to identify potential oyster aquaculture sites in Maine [36]. The RF model classifies benthic habitats based on camera observations of the sea floor at the Isles of Shoals, in the Gulf of Maine. This approach will allow for the modelling of Gulf of Maine kelp habitats across a greater portion of their depth range and over larger areas than traditional surveys can access, through the incorporation of variables that may govern substrate competition between kelps and red turf macroalgae.

## Methods

### Study site

The study site comprises a 10-by-13 km area around the Isles of Shoals. The center of the island group lies 20 km southeast of Portsmouth, New Hampshire, straddling the Maine-New Hampshire maritime border. It consists of eight islands and numerous smaller islets and rocks. The area was selected for this study because it has been the focus of several long-term subtidal ecology studies [13,14,16,17,37,38]. No approvals were required for our surveys within this area; subtidal areas are not privately owned and no collection or manipulation of animal life was performed.

### Satellite imagery

Data from the Thermal Infared Sensor onboard Landsat 8 and 9 is published by the U.S. Geological Survey at an interpolated 30 m resolution following atmospheric correction [35]. Studies validating these SST data tend to find <1°C differences between Landsat 8 imagery and buoy data [39,40].

Imagery from Landsat 8 was processed in Google Earth Engine [41,42]. Images containing the Isles of Shoals during the summer months of July through September between the years of 2019 and 2023 were isolated. The quality assurance band was used to mask cloud cover, and to eliminate images with greater than 25% local cloud cover around the Isles of Shoals – this step was taken to mitigate errors in cloud, cloud edge, and cloud shadow detection. This yielded a collection of 16 images: three from 2019, one from 2020, four from 2021, four from 2022, and four from 2023. The thermal infrared band was converted to degrees Celsius, and the collection of images was reduced to a raster containing the mean, median, standard deviation, and range of SST at each cell. Imagery from Landsat 9 was not included in analysis as this would result in further overrepresentation of the years 2022–2023 in the SST statistics.

### Bathymetry

Multibeam bathymetry around the Isles of Shoals was provided at a 4 m resolution by the Center for Coastal and Ocean Mapping/Joint Hydrographic Center [43]. The data were primarily collected between 2005 and 2010 using the R/V Coastal Surveyor, equipped with a Kongsberg EM3002D multibeam echosounder operating at 293 and 307 KHz. Some data along the outer fringes of the study area were collected between 2013 and 2014 using the NOASS Ferdinand R. Hassler, equipped with a Reson 7125 multibeam echosounder operating at 200 and 400 KHz.

Several variables were derived from this depth dataset. Slope, mean curvature, and slope aspect were calculated using the Spatial Analyst toolbox in ArcGIS Pro (version 2.9.0) [44]; slope aspect was further divided into northness and eastness of aspect by taking the cosine and sine of aspect in radians, respectively. A vector ruggedness measure (VRM) [45] was calculated with the Arc Hydro toolbox (version 2.9.111) [46], using a 3-cell (12 m) neighborhood. Slope, mean curvature, northness, eastness, and VRM have previously been used in benthic habitat classification as they relate to factors such as wave exposure and sedimentation [47,48]; these factors are contextually relevant to macroalgae community composition and kelp performance [49–52].

The Bathymetry- and Reflectivity-based Estimator of Seafloor Segments (BRESS, version 2.4.0) utility was used to classify the bathymetry into geomorphons [53,54]. Geomorphons describe kernels of homogenous terrain and have been used to characterize benthic habitats [55–57]. Bathymetry was split into six geomorphon types (flat, ridge, shoulder, slope, footslope, and valley) using a 2.5° flatness angle. As geomorphons are non-ordinal categorical data, the geomorphon types were one-hot encoded into six binary rasters (representing flat/non-flat area, shoulder/non-shoulder area, etc.). All bathymetric rasters were then downsampled to 30 m resolution to match the SST data.

### Variable collinearity

All rasters were clipped to a 2.5 km envelope around the islands using NOAA National Shoreline vector data [58]. Land was masked from the SST rasters with the depth raster, using a minimum depth of 1 m below sea level. Within the bathymetry dataset, Mean Lower Low Water is used as the vertical datum [43]; 1 m of additional depth removes all intertidal area from analysis. These steps were performed in ArcGIS Pro (version 2.9.0) using the Spatial Analyst, Analysis, and Data Management toolboxes [44].

Collinearity testing was performed on the ten continuous variables in the R programming language and computing environment (version 4.3.0) [59] with the inbuilt stats package, using ten thousand randomly selected cells and correlation coefficient thresholds of 0.7 and −0.7. Mean and median SST were found to be collinear (>0.7), and range and standard deviation of SST were found to be highly collinear (>0.9). Mean SST and range of SST were discarded, as they are more strongly affected by outliers than median SST and standard deviation of SST.

### Field surveys

**Fig 1:**
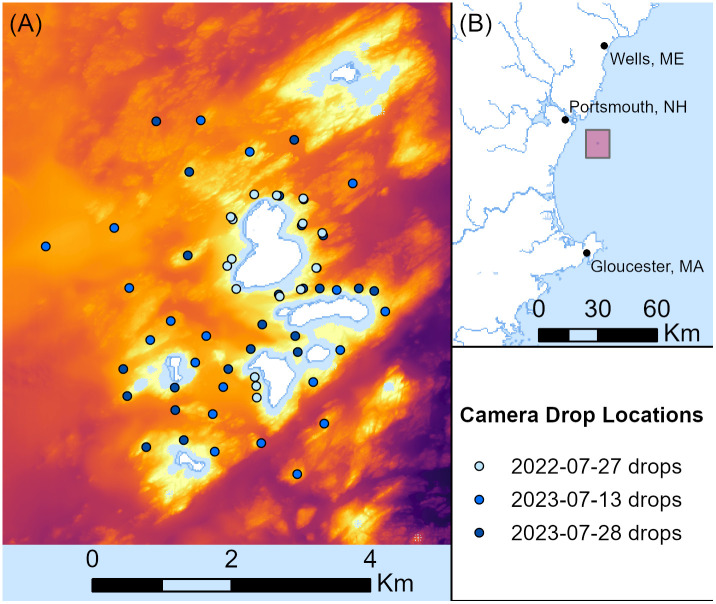
Isles of Shoals ground truth video acquisition. **(A)** Sampling locations overlaid on high-resolution bathymetry, 1:85,000 scale. **(B)** Isles of Shoals study area location, 1:3,000,000 scale. Landmasses are white areas outlined in blue. Basemap available from U.S. Geological Survey, National Geospatial Program.

Ground truth sampling was performed from a 6.7 m vessel on 27 Jul 2022, 13 Jul 2023, and 28 Jul 2023. Two GoPro HERO10 cameras were mounted along adjacent top edges of a metal frame (32 × 32 × 55 cm). While the cameras recorded video, the frame was lowered by line to the benthic surface and then retrieved. GPS coordinates from the vessel’s chart plotter were used to georeference the videos. The effects of currents on the vessel, rope, and frame were corrected by matching benthic landmarks in the recordings to the 4 m resolution bathymetry. Sixty-one drops were performed around the islands, to a maximum depth of 30 m (Fig 1). During deployment and retrieval, the vessel and camera frame drifted across the benthic surface. Combined with the wide horizontal field of view (approximately 200° across the two cameras), this often allowed observations to be made for 2–5 contiguous 30 m cells during each drop. Majority habitat types were identified among the resulting 247 observations. The observations were divided into four classes: bare substrate (79 observations), kelp-dominated habitat (77 observations), red turf-dominated habitat (45 observations), and an intermediate “mixed” macroalgae habitat (46 observations). In the context of this modelling approach, ‘habitat’ (e.g., ‘kelp habitat’ or ‘kelp-dominated habitat’) refers to areas either observed (in ground truth) or predicted (by RF models) to be primarily characterized by the corresponding cover type at a resolution of 30 meters, within the five years spanned by the SST data.

**Fig 2:**
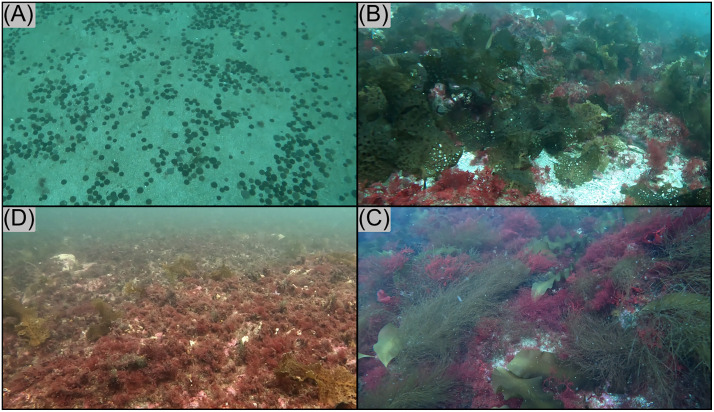
Examples of habitat types at the Isles of Shoals. **(A)** Bare substrate. **(B)** Kelp-dominated habitat. **(C)** Mixed macroalgae habitat. **(D)** Red turf-dominated habitat.

Observed kelp beds were primarily composed of *Saccharina latissima* or *Agarum clathratum*, with a transition from the former to the latter occurring with increasing depth. The presence of *D. japonica* could often be visually established within red turf assemblages (whether dominant or mixed with kelp), but most other local filamentous red macroalgae require collection and microscopy for confident species-level identification. Example images of these classes taken from drop camera footage from are provided in Fig 2.

### Random Forest modelling

RF models classify data by creating a “forest” of decision trees. Each tree is given a random subset of ground truth observations and divides them into increasingly pure nodes (in terms of class), selecting the best variable from a random subset of variables at each split [60]. RF classification methods have been used to characterize benthic habitats in bathymetry-based [32,33] and satellite imagery-based approaches [61–63]. Modelling was performed in R (version 4.3.0) using the randomForest and raster packages [59,64,65].

#### Monte Carlo simulation.

Though RF models are generally considered insensitive to overfitting, some researchers have demonstrated overfitting when assessing independent test datasets [66,67]. Monte Carlo simulation was employed to further minimize the risk of overfitting, to enable the computation of per-class F_1_ statistics, and to facilitate threshold-based mapping of habitat predictions. Within each simulation, a different integer was used to initialize pseudorandom number generation and divide ground truth observations into training (70%) and external validation (30%) datasets. A range of simulation counts between *n* = 50 and *n* = 300 was tested to determine a suitable number. Model performance stabilized around *n* = 160 simulations, indicated by diminishing improvements to model accuracy and variability. To provide a buffer, *n* = 175 was used.

#### Model assessment.

The primary values used to indicate model performance were training accuracy and validation accuracy. Training accuracy is the complement of the out-of-bag error value provided by the model (1 – Error_OOB_), validation accuracy is the accuracy of the trained model in classifying the external validation dataset kept outside of the model (in a given simulation). These values were averaged across the 175 Monte Carlo simulations for each model permutation. The difference between training and validation accuracy was used as an indicator of overfitting, with smaller differences suggesting a smaller likelihood of overfitting [68,69].

F_1_ scores and variable importances were also assessed. The F_1_ score was calculated for each habitat class on a one-versus-rest basis using precision and recall scores calculated using the external validation dataset. This score indicates the success of the model in distinguishing each habitat class. Variable importance was described in terms of Mean Decrease in Accuracy. This represents the average increase in error rate for a permutation of the model that does not include a given variable, i.e., more important variables represent a greater increase in error if removed from the model. Variable importance is calculated both on a per-class and model-wide basis.

#### Hyperparameter tuning.

The impacts of several RF hyperparameters were assessed to determine whether tuning them would further improve model accuracy. The first hyperparameter assessed was *ntree*, which determines the number of trees produced in an RF model. A range of *ntree* values between *n* = 50 and *n* = 350 were tested; diminishing improvements to model accuracy were achieved around *n* = 200 trees, so *n* = 215 was used to provide a buffer. The variable *mtry* determines the number of variables randomly subset and offered to each tree at each node split. Intermediate values are usually favored, as low values produce poor-performing trees and high-values produce homogenous trees (yielding a less-random forest). Values of *mtry* from *m* = 3 to *m* = 14 (with 14 total model variables) were tested, *m* = 6 was chosen. The hyperparameters *maxnodes*, the maximum number of nodes per tree, and *nodesize*, the minimum observation count of terminal nodes, were also assessed. Tuning these hyperparameters provided no further improvements to model accuracy, so the model defaults (unlimited *maxnodes; nodesize* of *n = *1) were used.

#### Raster classification.

The trained and tuned RF model was used to classify the habitats around the Isles of Shoals using the surface temperature and bathymetry rasters. This process yielded 175 classified rasters because of the Monte Carlo simulation process. A threshold-based map of habitat predictions was created from these rasters using the Spatial Analyst toolbox in ArcGIS Pro (version 2.9.0); for each cell, the class identified in the majority of rasters was identified, then assigned into a “high confidence” category if assigned in 75–100% of the rasters or a “low confidence” category if assigned in 50–75% of the rasters. The small proportion of cells with no majority class assignment were labeled as such. The threshold-based raster contains nine habitat classes: high- and low-confidence bare substrate, high- and low-confidence kelp-dominated habitat, high- and low-confidence mixed macroalgae habitat, high- and low-confidence red turf-dominated habitat, and the no-majority class. The depth limits of kelp habitats can vary locally based on abiotic factors that include substrate availability, temperature, and light availability [23,70]. As a maximum depth for the relevant macroalgae communities has not been established at the Isles of Shoals, the raster was masked to a generous 50 m depth limit.

## Results

### Model performance

The trained and tuned model achieved a mean training accuracy of 71.3% and a mean validation accuracy of 71.5%. The 0.2 percentage point difference between the accuracy values does not suggest overfitting. The mean precision, recall, and F_1_ score for each class are reported in Table 1. The F_1_ scores indicate that the model is better at classifying the bare substrate and kelp-dominated classes than the mixed macroalgae and red turf-dominated classes.

**Table 1:** Per-class precision, recall, and F_1_ score.

Class	Mean Precision	Mean Recall	Mean F_1_
**Bare Class**	0.805	0.745	0.770
**Kelp Class**	0.748	0.792	0.764
**Mixed Class**	0.671	0.631	0.635
**Red Class**	0.597	0.646	0.608

### Variable importance

The importance of each variable to each class and to the model overall are reported in Table 2. Depth, VRM, standard deviation of SST, median SST, slope, and the flat geomorphon were the most important variables to the model overall.

**Table 2:** Per-class and model-wide variable importance.

Variable	Bare Class	Kelp Class	Mixed Class	Red Class	Model
**Depth**	6.55	**14.55**	**5.91**	**15.38**	20.19
**VRM**	**14.41**	**9.34**	4.62	**6.08**	16.25
**Std Dev of SST**	5.60	7.17	**15.62**	**6.06**	16.00
**Median SST**	6.36	**11.65**	4.82	1.70	12.82
**Slope**	**10.65**	7.38	1.91	2.10	12.16
**Flat Geomorphon**	**8.40**	4.01	**6.16**	3.47	9.27
**Curvature**	7.52	−0.19	1.68	1.95	6.61
**Northness**	1.37	3.01	5.30	1.46	5.76
**Eastness**	1.59	4.90	1.00	1.49	4.99
**Slope Geomorphon**	4.30	1.34	1.41	2.02	4.75
**Ridge Geomorphon**	2.88	1.66	0.85	0.34	2.93
**Footslope Geomorphon**	1.42	0.30	1.19	2.52	2.51
**Shoulder Geomorphon**	−0.32	−0.18	0.19	0.68	0.19
**Valley Geomorphon**	0.42	0.16	−0.79	0.13	−0.11

Variable importances are reported in terms of Mean Decrease in Accuracy. Variables are ordered by mean model-wide importance; the three most important variables for each class are bolded, and the three least important are underlined.

VRM, slope, the flat geomorphon, and curvature were the most important to the bare substrate class. Depth, median SST, VRM, slope, and standard deviation of SST were the most important to the kelp-dominated class. Standard deviation of SST, the flat geomorphon, depth, and northness of aspect were the most important to the mixed macroalgae class. Depth, VRM, standard deviation of SST, and the flat geomorphon were the most important to the red turf-dominated class.

The contribution of eastness of aspect, and the ridge, slope, and footslope geomorphons were small. The shoulder geomorphon contributed only slightly to the model. The valley geomorphon had a small negative importance to the model, but this only became apparent after hyperparameter tuning. As hyperparameter values depend in part on the number of variables in the model, this variable was retained.

### Habitat classification

**Fig 3:**
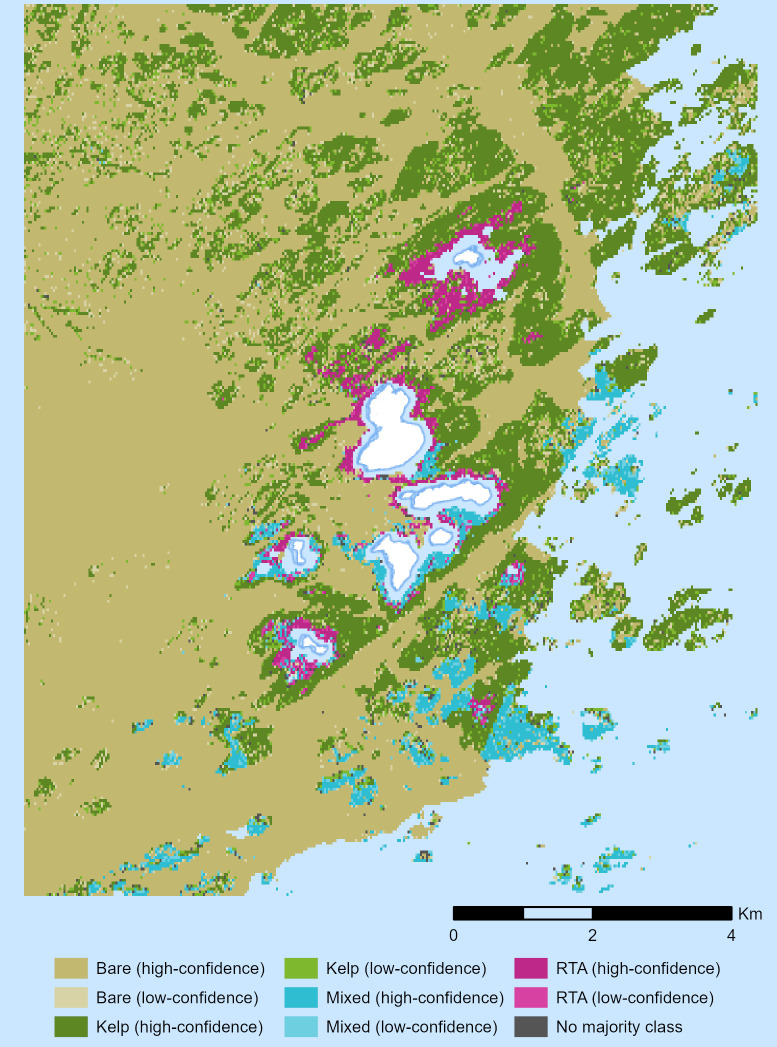
Threshold-based habitat classification at the Isles of Shoals. Predicted habitats at 30 m resolution, at 1:85,000 scale. “RTA” refers to red turf algae-dominated habitat. Basemap available from U.S. Geological Survey, National Geospatial Program.

The percentage of cells assigned to each class at each confidence level are reported in Table 3. The threshold-based habitat classification is presented in Fig 3.

**Table 3:** Predicted percent cover of habitats by confidence level.

Habitat Class	High-confidence	Low-confidence	Total Cover
**Bare Class**	61.7%	3.4%	65.1%
**Kelp Class**	23.3%	4.3%	27.6%
**Mixed Class**	3.1%	1.2%	4.3%
**Red Class**	1.7%	0.6%	2.3%
**No-majority Class**	--	--	0.8%

Bare substrate was the most common class predicted around the Isles of Shoals, comprising 65.1% of total cells. Kelp-dominated habitat predictions were the second most common, comprising 27.6% of total cells and 80.7% of macroalgae (i.e., non-bare) habitat predictions. Mixed macroalgae habitat and red turf-dominated habitat comprise 4.3% and 2.3% of total cells, and 12.7% and 6.7% of macroalgae habitat predictions respectively.

More than two thirds of predictions were made above the high-confidence threshold for each class. 94.8% of bare substrate predictions, 84.6% of kelp-dominated habitat predictions, 71.3% of mixed macroalgae habitat predictions, and 73.5% of red turf-dominated habitat predictions were made at the high-confidence threshold. Overall, 89.8% of cells were high-confidence predictions, 9.5% of cells were low-confidence predictions, and 0.8% of cells had no majority class assigned.

## Discussion

Bathymetric variables, namely slope, VRM, flat geomorphon, and curvature, were highly important in distinguishing bare substrate from the three macroalgae habitat classes. Most flat areas observed around the Isles of Shoals are composed of sediments that are not suitable substrate for the relevant macroalgae habitats, and each of these variables aid in distinguishing flat and non-flat areas. Median SST and standard deviation of SST were more important in distinguishing between the macroalgae classes than dividing them from the bare substrate class. Depth was a strong separator of kelp-dominated habitat (deeper) from red turf-dominated habitat (shallower), though somewhat less important for intermediate mixed macroalgae habitat, which occurred across a range of depths. The importance of depth and VRM in benthic habitat characterization is consistent with past bathymetric modelling [33]. Beyond the identification of flat, unsuitable areas, slope affects the likelihood of sediment accumulation [47,48].

Satellite-based models have predicted kelp habitat at finer resolutions and with greater accuracy [30], but are strongly constrained by depth. Using optical imagery from SPOT satellites, researchers modelled kelp habitat in the Gulf of St. Lawrence with ~90% accuracy at a 1 m resolution, down to 7 m depth [30]. Similar modelling of seagrass beds by in Nova Scotia was restricted to 8 m depth at a 1.5 m resolution [71]. The area of study at the Isles of Shoals comprises approximately 132 km^2^ (at the initial 4 m bathymetry resolution); areas between 1–8 m depth account for 1.2 km^2^ of this area, while areas down to the 50 m depth threshold account for 102 km^2^. This represents up to an 85-fold increase in modelled habitat. Ground truth sampling was performed only to 30 m depth (representing 45.8 km^2^ of modelled habitat, a 38-fold increase) due to practical constraints; deeper sampling could further validate the habitat predictions between 30–50 m and provide a clearer depth cutoff for the relevant macroalgae communities.

Bathymetry-based models are unconstrained by optical depth and have also outperformed this model [48], but regional ecological factors may reduce their suitability in this context. Researchers achieved 87% accuracy modelling similar categories of macroalgae and invertebrate habitats at a 2.5 m resolution in Victoria, Australia using derivatives of bathymetry and backscatter data [48]. Within their model, however, estimates of the kelp-dominated habitat classes were less reliable than the overall model accuracy (~62% compared to 87%) and less reliable than kelp habitat predictions in our model. Bathymetry- and backscatter-based modelling may be more appropriate in southern Australia than in the Gulf of Maine due to the difference in stressors to kelp habitat between the two regions; previous research identifies sedimentation and eutrophication as key *Ecklonia radiata* stressors in southern Australia [72], while species invasion and rising ocean temperatures are thought to be primary drivers of *S. latissima* decline in New England [15,73,74].

Some sources of model error may be fundamental to the ecology of the system. Establishing distinct classes in an ecosystem where a continuum of species exists leads to fuzzy boundaries between the classes. Researchers identifying shallow benthic habitats from optical imagery addressed this by prioritizing seagrass over other habitat types; where seagrass and macroalgae were present, the seagrass class was used [71]. We chose to retain the mixed macroalgae class rather than wholly prioritize kelp identification due to the distinct patch size and habitat heterogeneity characteristics previously observed within that habitat type at the Isles of Shoals [73]. A further constraint on accuracy is the 16-day revisit time of Landsat 8, which is too infrequent to capture the duration, intensity, or potentially even occurrence of marine heatwaves. These heatwaves can reduce kelp performance [20], impeding propagule production and competition for space with introduced species such as the heat-tolerant, vegetatively-reproducing *D. japonica* [75–77]. Sources that offer SST data over shorter intervals, such as MODIS, are able to capture these warming events, but the spatial resolution of these datasets (typically at scales of 1 km or more) are not suited for habitat modelling at the scales we are interested in. We also expect that there are biotic and abiotic factors (such as grazer density or storm intensity) that do not lend themselves to quantification in raster format.

## Conclusion

RF classification utilizing acoustic bathymetry and satellite-derived SST data predicted benthic habitats around the Isles of Shoals with a mean accuracy of 71%; the F_1_ score for the kelp habitat class was 0.770, indicating greater accuracy in predicting this class. Within our study area, the most important variables for kelp habitat classification were the bathymetry-derived variables of depth, slope, and VRM, and the satellite imagery-derived variables of median SST and standard deviation of SST.

This approach provides advantages over several existing monitoring and modelling methods. It predicts benthic habitat types over a much greater area than can be covered by traditional SCUBA surveys and accesses a greater portion of the potential depth range of kelp habitats than modelling approaches using optical imagery. SST variables were highly important in describing kelp habitat, suggesting that within the Gulf of Maine, an integrative approach combining SST and bathymetry will provide better results than approaches using only bathymetry data. Our model utilizes a modern SST data source that is largely untapped within benthic habitat modelling. The use of median and standard deviation SST values mitigates the long revisit time of the Landsat satellites by reducing the impact of outlier values. Landsat SST data allows for habitat predictions to be made at spatial resolutions over 30 times more fine than past approaches that integrated both SST and bathymetry data [34]. We also introduce a lower-effort method of ground truth data collection than is used in many studies; despite the 13 km between our pier in New Castle and our study site, only 12 hours of vessel time were needed for sampling.

Future research should test the generalizability of this approach to larger and more distant areas. Further sampling at spatially independent sites will indicate how applicable the model itself is to the Gulf of Maine. Variables such as northness and eastness, while ranked among the lower half in model-wide variable importance, relate here to the spatial geometry of the Isles of Shoals and their importance and meaning may change at other sites; in such cases it may be appropriate to remove these variables or retrain the model locally. Annual collection of habitat data in the area can be used to assess the accuracy of this modelling approach over time, in order to determine how frequently model recalibration ought to be performed.

Monitoring the extent of subsurface kelp beds is crucial in many regions around the world where these habitats are currently threatened. Refinement of our modelling approach and broadening of its spatial extent to larger portions of the Gulf of Maine, as well as expansion upon the predictor variables identified as important by our models, may help identify conditions under which kelp habitats are more likely to remain stable over the near future. Ecosystem based management models can integrate these results, along with historical knowledge, to prioritize sites for restoration, marine spatial planning, and kelp aquaculture. One area currently identified as a hotspot for *S. latissima* density and stability in the Gulf of Maine is Cashes Ledge [21]. This area is currently protected by the New England Fisheries Management Council, and has been nominated for permanent protection under National Marine Sanctuary status – it would be the second in the Gulf of Maine after Stellwagen Bank National Marine Sanctuary [78]. Identification of additional areas through modelling methods would allow for more protection of these biodiverse habitats.
